# iPACK block (local anesthetic infiltration of the interspace between the popliteal artery and the posterior knee capsule) added to the adductor canal blocks versus the adductor canal blocks in the pain management after total knee arthroplasty: a systematic review and meta-analysis

**DOI:** 10.1186/s13018-022-03272-5

**Published:** 2022-08-12

**Authors:** Jiao Guo, Minna Hou, Gaixia Shi, Ning Bai, Miao Huo

**Affiliations:** 1 Department of Anesthesiology, Shaanxi Provincial People’s Hospital, No. 256 Youyi Xi Road, Xi’an, China; 2 Department of Nursing, Shaanxi Provincial People’s Hospital, Xi’an, China

**Keywords:** iPACK block, Adductor canal block, Total knee arthroplasty, Meta-analysis, GOSH analysis, Meta-regression

## Abstract

**Background:**

Several studies have suggested that the addition of iPACK block (the popliteal artery and the posterior knee capsule have been given interspace local anesthetic infiltration) might get better analgesia than adductor canal block (ACB) only after total knee arthroplasty (TKA). This paper compiles all available evidence on the effect of two analgesia regimens (ACB and iPACK + ACB) involving all sides.

**Methods:**

We searched in eight major databases for all clinical trials discussing the effect of two analgesia regimens after TKA. Statistical analyses were conducted by Stata and RevMan Software. In addition, we performed GOSH analysis, subgroup analysis, meta-regression analysis to study the source of heterogeneity. Publication bias was checked using Egger’s test. Trim-and-fill analysis was applied in terms of sensitivity analysis of the results.

**Results:**

There are fourteen eligible studies for our meta-analysis. There are significant differences between the two groups in VAS score at rest and with activity, and the VAS scores were lower in the ACB + iPACK Group (VAS scores at rest: 95%CI [− 0.96, − 0.53], *P* < 0.00001. VAS scores with activity: 95%CI [− 0.79, − 0.43], *P* < 0.00001). A differential was discovered to support the ACB + iPACK Group when comparing the two groups on postoperative cumulative morphine consumption (95%CI: [− 0.52, − 0.14], *P*: 0.0007). The patients in the group of ACB + iPACK performed better in the postoperative range of knee movement (95%CI: [5.18, 10.21], *P* < 0.00001) and walking distance (95%CI: [0.15, 0.41], *P* < 0.00001). There were significant differences between the patients in the ACB + iPACK Group and ACB Group on the TUG test of POD1 and POD2. We found that patients' hospital stays in the ACB + iPACK Group were significantly shorter than in the ACB Group (95%CI: [− 0.78, − 0.16], *P*: 0.003). No difference was found between the patients in the ACB + iPACK Group and ACB Group on postoperative quadriceps muscle strength and the incidence of PONV.

**Conclusion:**

The addition of iPACK lowers postoperative VAS scores, cumulative morphine consumption, and hospital stays. Meanwhile, the addition of iPACK improves postoperative patients’ activity performance without extra side effects. iPACK combined with ACB proves to be a suitable pain management technique after TKA.

## Introduction

Total knee arthroplasty (TKA) refers to a viable treatment asymptomatic osteoarthritis of the knee refractory to conservative measures. According to the estimated project by the year 2030, 3.48 million TKAs will have been conducted on a yearly basis [1]. However, relieving postoperative pains, following total knee arthroplasty is of vitally important to the postoperative recovery of the patients.

Currently, ultrasound-guided adductor canal blocks (ACBs) perform an adjunct with multimodal pain protocol on the patients with TKA effectively minimize postoperative pain and narcotic consumption [2]. Adductor canal block (ACB) is ever contributing an approach to femoral nerve block after TKA. ACB is usually conducted under ultrasound machines and local anesthetic is injected nearby the saphenous nerve in the adductor canal [3, 4]. However, ACB cannot lead to a relieved posterior knee pain [5, 6]. The recent ultrasound technique instructed local anesthetic infiltration of the interspace between the popliteal artery and the posterior knee capsule (iPACK block) has offered dramatic posterior knee analgesia. Accordingly, a number of clinic doctors chose to make additional iPACK block to ACB to control postoperative pain. The ultrasound transducer is laid for identifying the femoral condyle. After identifying the popliteal artery, the tip of needle is placed at the right middle part in the bone and the popliteal artery. Local anesthetic is injected at that spot [7]. But it is controversial that whether iPACK block should add to the analgesia regimen (ACB included) in the patients after TKA.

This research aims at a systematical review over the literature for ascertaining if iPACK block is able to take in extra analgesic advantage for present multimodal analgesia regimens after TKA. We compiled all available evidence on the effect of these two analgesia regimens (ACB and iPACK + ACB) involving postoperative pain score, postoperative muscle strength, postoperative rehabilitation training, and perioperative adverse effects.

## Methods

### Inclusion and exclusion criteria

This research provides a system-based review based meta-analysis oriented with randomized controlled trials (RCTs) for comparing the effects which two analgesia regimens (ACB and iPACK + ACB) can exert after TKA. The published contents abide by the PRISMA Statement [8].

The PICO framework formulated the review question. The research illustrated the discussion on (Population) adult patients who had TKA (Intervention vs. Comparator) in combination to the analgesia regimen iPACK + ACB versus with the analgesia regimen ACB and measure (Outcome) postoperative pain score, postoperative muscle strength, postoperative adverse effect and postoperative rehabilitation training.

Our primary outcomes include postoperative pain score at 8-h phase, 12-h phase, 24-h phase, 48-h phase and discharge (at rest and with activity), postoperative morphine consumption, postoperative quadriceps strength, postoperative range of knee movement (ROM), postoperative walk distance, Timed Up and Go (TUG) test, hospital stays, and the incidence of postoperative nausea and vomiting (PONV).

We used the visual analog scale (VAS) for the pain score. For postoperative morphine consumption, we collected postoperative consumption (mg) of each study on the first and second day after surgery and the total consumption. The hospital stays were calculated in hours. The postoperative walking distance was in meters, and the walking distance of postoperative on the first day, the second day, and accumulated in each study was collected. The incidence of PONV was based on the occurrence of nausea or vomiting symptoms. Our study separately collected the postoperative ROM on the first, second, and third day after the surgery. The postoperative ROM is based on the range of the extension and flexion of the knee. Similarly, our study collected the results of TUG tests of patients on the first day after the surgery, the second day after the surgery, and at discharge. The TUG test measures the time it takes a patient to rise from a chair, walk 3 m, and return to the same chair without physical assistance [9]. Our study collected patients' quadriceps muscle strength in each study on the first day and the second day after the surgery. And manual muscle testing scores assessed quadriceps strength, and the grading was recorded from 0 to 5 [10].

### Search, selection, and data extraction

Our group investigated electronic databases, which contained the English database (PubMed, Embase, Cochrane Library, Web of Science, ClinicalTrials.gov) and the Chinese database (CNKI, WanFang Data, CQVIP). The following terms were used to search for relevant records: “iPACK,” “iPACK block,” “iPACK nerve block,” “adductor canal block,” “perioperative adductor canal block,” “adductor canal blockade,” “ACB,” “total knee replacement (TKA),” “Arthroplasties, Replacement, Knee,” “Arthroplasty, Knee Replacement,” “Knee Replacement Arthroplasties,” “Knee Replacement Arthroplasty,” “Replacement Arthroplasties, Knee,” “Knee Arthroplasty, Total,” “Arthroplasty, Total Knee,” “Total Knee Arthroplasty,” “Replacement, Total Knee,” “Total Knee Replacement,” “Knee Replacement, Total,” “Knee Arthroplasty,” “Arthroplasty, Knee,” “Arthroplasties, Knee Replacement,” “Replacement Arthroplasty, Knee,” “Arthroplasty, Replacement, Partial Knee,” “Unicompartmental Knee Arthroplasty,” “Arthroplasty, Unicompartmental Knee,” “Knee Arthroplasty, Unicompartmental,” “Unicondylar Knee Arthroplasty,” “Arthroplasty, Unicondylar Knee,” “Knee Arthroplasty, Unicondylar,” “Partial Knee Arthroplasty,” “Arthroplasty, Partial Knee,” “Knee Arthroplasty, Partial,” “Unicondylar Knee Replacement,” “Knee Replacement, Unicondylar,” “Partial Knee Replacement,” “Knee Replacement, Partial,” “Unicompartmental Knee Replacement,” “Knee Replacement, Unicompartmental.” We used the Boolean operator “OR” or “And” to connect these terms. Two experts used unified Microsoft Excel to collate the data independently. In case of inconsistencies, it was decided by the third expert.

### Risk of bias (RoB) assessment

Coupled with crucial points in methodology (PH, GV, IP, and IT), the Cochrane Risk of Bias Tool was employed for rating the Risk of Bias [11]. The results of the RoB estimation were combined with findings illustrations, instead of integrating into statistical analysis. When a consensus was reached, disparities from the estimation were addressed.

### Quality of evidence

Such approaches as Grading of Recommendations, Assessment, Development and Evaluation (GRADE) were employed for rating the quality featured by evidence on every outcome.

### Statistical analysis

In virtue of RevMan and Stata Software, the statistical analysis was committed by an expertised statistician. Besides, evaluation was made on the pooled relative risks (RRs) based on 95% confidence intervals (CIs) over the total preliminary outcomes.

Statistical analyses were merely conducted on the condition of the availability least-wise two RCTs in each group. Because the research setting cannot make an exact match, a random effect model was implemented based on the DerSimonian–Laird estimation (11). *I*^2^ and chi^2^ tests, employed for qualifying statistical heterogeneity, aimed at *P*-values individually; P < 0.1 marked a dramatic heterogeneity [12]. When the heterogeneity was significant (*I*^2^ > 65%), we performed GOSH analysis and subgroup analysis to study the source of heterogeneity. In addition, publication bias was checked using Egger’s test. Trim-and-fill analysis was applied in terms of sensitivity analysis of the results.

## Results

### Identifications and characteristics of the researches

**Fig. 1:**
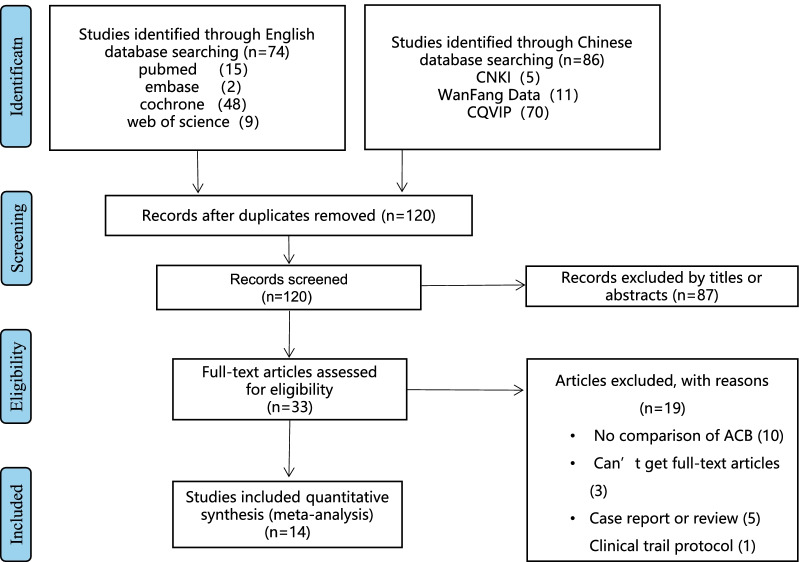
The flowchart of meta-analysis

Figure 1 manifests the flowchart included in the meta-analysis of our group and elimination reasons. Finally, 14 studies were contained in the meta-analysis [13–26]. Besides, the study was featured by the summarization in Table 1. Table1 shows that these 14 clinical trial designs have many discrepancies: 1. Some experiments used general anesthesia [11, 13, 14, 16, 18, 20, 21, 24], while others adopted spinal anesthesia [12, 15, 17, 19, 22, 23]. 2. Some researchers performed nerve blocks before the surgery [11–16, 18, 20–22], while others performed nerve blocks after the surgery [15, 19, 23, 24]. 3. Some clinical trials used multimodal analgesia and placed postoperative analgesia pumps [12–14, 18, 21, 22], while others did not use it.

**Table 1:** Characteristics of the study included

Author, Year	Study design	Anesthesia and block timing	Composition of interventions	Composition of controls	Perioperative analgesia strategy	Primary outcome
DongHai Li, 2020	RCT	General anesthesia (pre-operation)	*ACB + iPACK***:** ACB (20 ml of 0.2% ropivacaine, 2.0 mg/mL of epinephrine) iPACK (20 ml of 0.2% ropivacaine, 2.0 mg/mL of epinephrine)	*ACB *(20 ml of 0.2% ropivacaine, 2.0 mg/mL of epinephrine)	*Postoperative***:** ice compression devices; loxoprofen 60 mg PO bid; alprazolam 0.4 mg PO qd	Pain score (VAS score/morphine consumption) Ambulation ability(the knee flexion angle/extension angle/quadriceps strength/patients’ daily ambulation distance/knee function KSS/WOMAC physical function/TUG test)
Jason Ochroch, 2020	RCT	Spinal anesthesia (pre-operation)	*ACB + iPACK***:** ACB (20 ml of 0.5% ropivacaine) iPACK (20 ml of 0.5% ropivacaine)	*ACB* (20 ml of 0.5% ropivacaine)	*Preoperative***:** Acetaminophen 1000 mg PO; Gabapentin 300 mg PO; Celecoxib 200 mg PO *Postoperative***:** Adductor canal catheter, ropivacaine 0.2% 8 mL/hour with demand bolus of 5 mL, lockout interval 30 min × 2 days; Acetaminophen 1000 mg PO every 8 h × 3 days; Celecoxib 200 mg PO every 12 h × 3 days; Gabapentin 300 mg PO every 12 h × 7 days; Oxycodone 5–10 mg PO every 4 h PRN	Pain score (Opioid consumption/Presence of posterior knee pain) The quality of pain management (American Pain Society Patient Outcome Questionnaire) Ambulation ability (ambulation distance/TUG test)
Ling Hu, 2020	RCT	General anesthesia (pre-operation)	*ACB + iPACK***:** ACB (20 ml of 0.2%ropivacaine) iPACK (15 ml of 0.2% ropivacaine)	*ACB* (25 ml of 0.375% ropivacaine)	*Postoperative* PCA (sufentanil 1ug/ml, background dose 2 ml/h, lockout interval 15 min × 2 days)	Pain score (VAS score/times of Intravenous parecoxib) Ambulation ability (the range of movement/time of first ambulation after operation)
Matthew E. Patterson, 2020	RCT	General anesthesia (pre-operation)	*ACB + iPACK* ACB (20 ml of 0.25% ropivacaine with epinephrine 3 mg/mL) iPACK (15 ml of ropivacaine 0.25% with epinephrine 3 mg/mL)	*ACB* (20 ml of 0.25% ropivacaine with epinephrine 3 mcg/mL)	*Preoperative* Pregabalin 150 mg PO *Postoperative* Adductor canal catheter, ropivacaine 0.2% 8 mL/h × 2 days; Acetaminophen 1 g PO every 8 h while in hospital; Celecoxib 400 mg PO daily while in hospital; Gabapentin 150 mg PO every night while in hospital	Pain score (Pain scale scores at rest and during physical therapy/ opioid consumption) Ambulation ability (Walk distance)
Min Li, 2019	RCT	Spinal anesthesia (post-operation)	*ACB + iPACK* ACB (20 ml of 0.33% ropivacaine) iPACK (15 ml of 0.33% ropivacaine)	*ACB* (20 ml of 0.33% ropivacaine)	*Preoperative* flurbiprofen axetil 50 mg IV *Postoperative* celecoxib 200 mg PO bid;	Pain score (NRS score/ nalbuphine consumption) Ambulation ability (ambulation distance/maximal knee flexion)
QiuRu Wang, 2020	RCT	General anesthesia (pre-operation)	*ACB + iPACK* ACB (20 ml of 0.2% ropivacaine with epinephrine 2ug/mL) iPACK (20 ml of ropivacaine 0.2% with epinephrine 2ug/mL)	*ACB* (20 ml of 0.2% ropivacaine with epinephrine 2ug/mL)	*Preoperative* celecoxib 200 mg PO bid; *Postoperative* ice compression devices; celecoxib 200 mg PO bid; oxycodone 10 mg PO bid	Pain score (VAS score/ morphine consumption) Ambulation ability (maximal knee flexion/ambulation distance/Muscle force)
R. Tak, 2020	RCT	Spinal anesthesia (unclear)	*ACB + iPACK* ACB (20 ml of 0.2% ropivacaine) iPACK (20 ml of 0.2% ropivacaine)	*ACB* (20 ml of 0.2% ropivacaine)	*Preoperative* Celecoxib 200 mg PO; Gabapentin 300 mg PO *Postoperative* paracetamol 1 g IV tid × 3 days; afterward paracetamol 1 g PO tid; Gabapentin 300 mg PO qd × 4 weeks	pain score (VAS scores/opioid consumption) Ambulation ability (ambulation distance/TUG test, 30 s chair stand test/sitting active extension lag test/maximal knee flexion)
Li Shen, 2019	RCT	General anesthesia (pre-operation)	*ACB + iPACK* ACB (25 ml of 0.375% ropivacaine) iPACK (30 ml of 0.2% ropivacaine)	*ACB* (25 ml of 0.375% ropivacaine)	*Postoperative* PCA (sufentanil 1ug/ml, background dose 2 ml/h with demand bolus of 4 ml, lockout interval 30 min × 2 days)	Pain score (VAS score/ sufentanil consumption/Press times of PCA) Muscle force (Bromage score) Ambulation ability (maximal knee flexion/time of off-bed/the time of first straight leg raising)
S. R. Sankineani, 2018	non-RCT	Spinal anesthesia (post-operation)	*ACB + iPACK* ACB (20 ml of 0.2%ropivacaine) iPACK (15 ml of 0.2% ropivacaine)	*ACB* (20 ml of 0.2%ropivacaine)	*Preoperative* Celecoxib 200 mg PO; Gabapentin 300 mg PO *Postoperative* paracetamol 1 g IV tid × 3 days; afterward paracetamol 1 g PO tid; Gabapentin 300 mg PO qd × 4 weeks	Pain score (VAS score) Ambulation ability (ambulation distance/ the range of movement)
XingFeng Zhou, 2020	RCT	General anesthesia (pre-operation)	*ACB + iPACK* ACB (25 ml of 0.25%ropivacaine) iPACK (30 ml of 0.25% ropivacaine)	*ACB* (25 ml of 0.25%ropivacaine)	*Postoperative***:** PCA (sufentanil 1ug/ml × 2 days)	Pain score (VAS score) Ambulation ability (maximal knee flexion/ the time of first straight leg raising/time of off-bed)
YuQuan Li, 2020	non-RCT	General anesthesia (pre-operation)	*ACB + iPACK* ACB (30 ml of 0.375% ropivacaine) iPACK (30 ml of 0.2% ropivacaine)	*ACB* (30 ml of 0.375% ropivacaine)	*Postoperative* PCA (sufentanil 1ug/ml, background dose 2 ml/h with demand bolus of 4 ml, lockout interval 30 min × 2 days)	Pain score (VAS score/ Press times of PCA) Ambulation ability (Bromage score)
Chutikant Vichainarong 2020	RCT	Spinal anesthesia (pre-operation)	*CACB + iPACK + LIA* CACB (20 mL of 0.25% levobupivacaine. Levobupivacaine 0.15% was continuously dripped at 5 mL/hour via pump) iPACK (20 mL of 0.25% levobupivacaine) LIA (levobupivacaine 100 mg, ketorolac 30 mg, epinephrine 0.3 mg diluted to a total volume of 80 mL)	*CABA + LIA* CACB (20 mL of 0.25% levobupivacaine. Levobupivacaine 0.15% was continuously dripped at 5 mL/hour via pump) LIA (levobupivacaine 100 mg, ketorolac 30 mg, epinephrine 0.3 mg diluted to a total volume of 80 mL)	*Preoperative* Acetaminophen 650 mg PO; Celecoxib 400 mg PO *Postoperative* CABA;15 mg ketorolac IV; 650 mg acetaminophen PO. q6h; 400 mg Celebrex PO. half a tablet of tramadol hydrochloride/acetaminophen PO. daily If patients presented with persisting pain,2 mg of intravenous morphine as rescue therapy	Morphine consumption within 24 h numerical rating scale pain scores incidence of posterior knee pain performance test results patient satisfaction length of stay adverse events
Tayfun Et 2022	RCT	Spinal anesthesia (post-operation)	*ACB + iPACK* ACB (20 mL of 0.5% bupivacaine) iPACK (20 mL of 0.5% bupivacaine)	*ACB* (20 mL of 0.5% bupivacaine)	*Preoperatively* Acetaminophen 1000 mg PO; diclofenac sodium 75 mg PO *Postoperatively* acetaminophen (1 g IV every 6 h, 4 doses); diclofenac (50 mg PO. tid, 25 mg for ≥ 75 years of age); tramadol 100 mg IV as a rescue analgesia when patient complained of pain with NRS > 4	the area under the curve (AUC) numeric rating scale (NRS) at 48 h cumulative postoperative analgesic consumption within 48 h Timed Up and Go test range of motion length of hospital stays patient satisfaction adverse events
Ping Mou 2021	RCT	General anesthesia (post-operation)	*ACB + iPACK*: ACB (20 ml of 0.25% ropivacaine, 2.0 ug/mL of epinephrine) iPACK (20 ml of 0.25% ropivacaine, 2.0 ug/mL of epinephrine)	*ACB* (20 ml of 0.25% ropivacaine, 2.0 ug/mL of epinephrine)	*Preoperatively* celecoxib 200 mg PO *Postoperatively* A cold pack was used to decrease pain; celecoxib (200 mg, PO. twice daily); pregabalin (150 mg, PO. twice daily); Oxycodone hydrochloride tablet (10 mg) was reserved as secondary rescue analgesia	postoperative pain scores opioid consumption functional evaluation postoperative complications

*PO* Take orally, *tid* Three times a day, *qd* Four times a day, *bid* Twice a day, *ACB* Ultrasound-guided adductor canal blocks, *iPACK* ultrasound-guided local anesthetic infiltration of the interspace between popliteal artery and the capsule of posterior knee

### RoB, publication bias, and sensitivity analysis

**Fig. 2:**
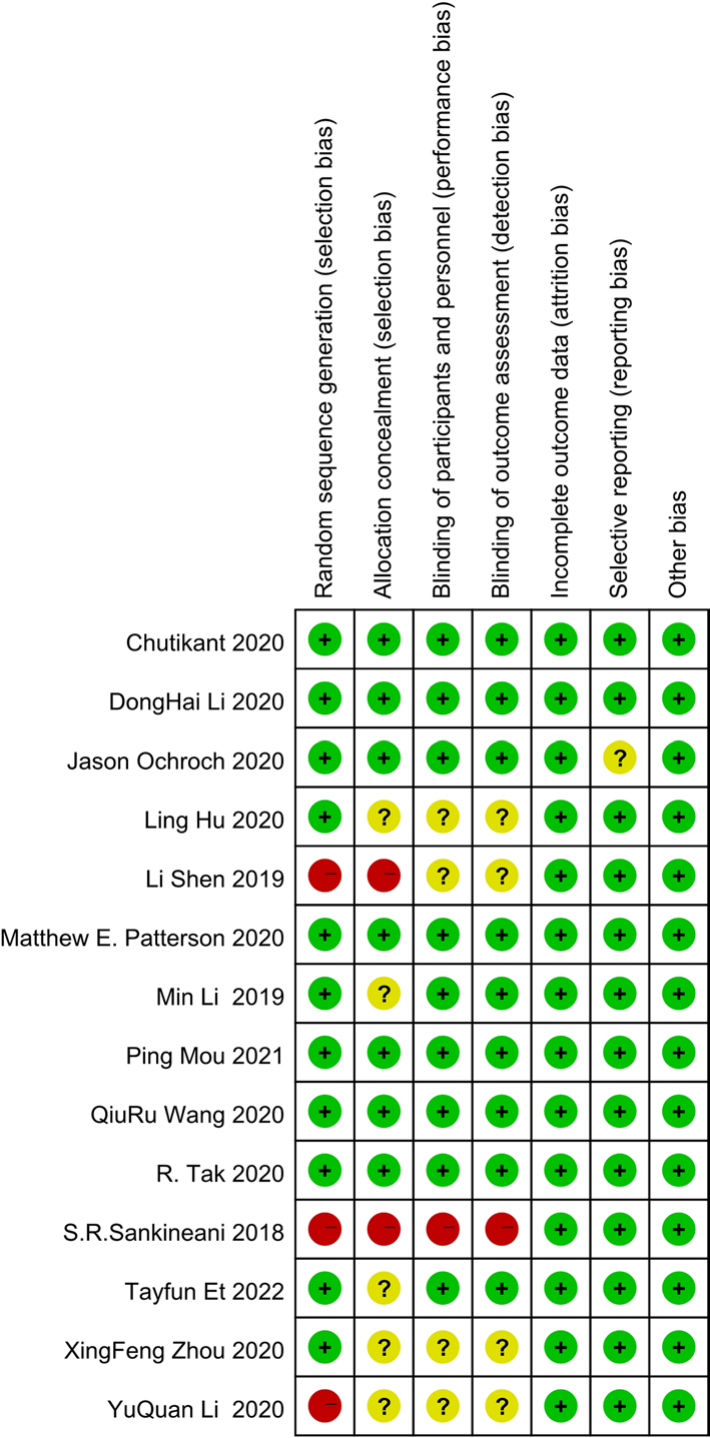
Risk of bias table: “ + ”: low risk of bias; “?”: unclear risk of bias; and “ − ”: high risk of bias

Despite the scarcity of selective reporting, some aspects could not suit the criteria of low RoB, including random sequence generation, hidden allocation, blinding in addition to selective reporting. Three fourteenths of these researches were regarded as highly risky items. The summarization of RoB includes RCTs in Fig. 2. We performed statistical analyses, publication bias checking, and sensitivity analysis of all results. The results of the heterogeneity test, publication bias, and trim-and-fill analysis are summarized in Table 2*.*

**Table 2:** *Results* of heterogeneity test, publication bias, and trim-and-fill analysis

Outcomes	No. of studies	Sample size	iPACK + ACB vs ACB	*I*^2^ (%)	*P*	Publication bias (Egger's test)				Trim-and-fill Analysis
			Mean difference 95%CI			*t*	*P*	95%CI		
VAS score at rest (8 h post-op)	7	614	− 1.08 [− 1.47, − 0.70]	89	< 0.0001	− 1.45	0.206	− 46.86	13.03	Stable
VAS score at rest (12 h post-op)	8	640	− 1.00 [− 1.43, − 0.58]	86	< 0.0001	− 2.76	0.033	− 22.31	− 1.33	Stable
VAS score at rest (24 h post-op)	9	754	− 0.56 [− 0.93, − 0.20]	89	0.002	− 1.57	0.16	− 27.52	5.55	Stable
VAS score at rest (48 h post-op)	10	874	− 0.62 [− 0.96, − 0.26]	93	0.0007	− 3.08	0.015	− 20.55	− 2.93	Stable
VAS score at rest (at discharge)	2	180	− 0.10 [− 0.28, 0.09]	0	0.31	NA	NA	NA	NA	Stable
VAS score at rest (overall)	10	3062	− 0.75 [− 0.96, − 0.53]	94	< 0.0001					
VAS score with activity (8 h post-op)	4	300	− 0.83 [− 1.04, − 0.63]	0	< 0.0001	0.03	0.98	− 27.98	28.34	stable
VAS score with activity (12 h post-op)	6	460	− 0.72 [− 1.09, − 0.34]	79	0.0002	− 1.52	0.204	− 28.86	8.47	stable
VAS score with activity (24 h post-op)	6	460	− 0.53 [− 0.59, − 0.11]	79	0.01	− 2.04	0.111	− 27.05	4.13	Stable
VAS score with activity (48 h post-op)	6	460	− 0.61 [− 1.00, − 0.23]	78	0.002	− 2.79	0.049	− 26.04	− 0.07	Stable
VAS score with activity (at discharge)	2	180	− 0.15 [− 0.37, 0.07]	0	0.18	NA	NA	NA	NA	Stable
VAS score with activity (overall)	6	1860	− 0.61 [− 0.79, − 0.43]	76	< 0.0001					
Cumulative morphine consumption (mg)	5	439	− 0.33 [− 0.52, − 0.14]	0	0.0007	− 0.56	0.617	− 15.96	11.21	Stable
ROM on POD1 (degree)	6	410	8.60 [5.13, 12.07]	76	< 0.0001	2.36	0.078	− 2.49	30.55	Stable
ROM on POD2 (degree)	7	550	8.04 [3.64, 12.44]	90	0.0003	1.35	0.233	− 9.54	30.82	Stable
ROM on POD3 (degree)	4	324	5.56 [0.37, 10.76]	84	0.04	4.38	0.048	0.23	24.62	Stable
ROM (overall)	9	1284	7.69 [5.18, 10.21]	86	< 0.0001					
TUG test on POD1 (s)	4	353	− 3.32 [− 5.57, − 1.06]	18	0.004	− 0.85	0.487	− 23.08	15.49	Stable
TUG test on POD2 (s)	3	269	− 6.49 [− 12.02, − 0.96]	58	0.02	− 0.37	0.775	− 131.5	124.09	Stable
TUG test at discharge (s)	2	180	− 0.88 [− 4.20, 2.45]	0	0.61	NA	NA	NA	NA	Stable
TUG test (overall)	5	832	− 3.63 [− 5.74, − 1.52]	43	0.0008					
walk distance on POD1 (meters)	5	439	0.23 [0.04, 0.41]	0	0.02	2.27	0.107	− 2.21	13.3	Unstable
Walk distance on POD2 (meters)	4	320	0.21 [− 0.01, 0.43]	0	0.06	5.01	0.038	1.1	14.47	Stable
Postoperative cumulative walk distance (meters)	2	233	0.48 [0.15, 0.82]	38	0.004	NA	NA	NA	NA	Stable
Walk distance (overall)	7	992	0.28 [0.15, 0.41]	2	< 0.0001					
Quadriceps muscle strength on POD1	4	320	− 0.11 [− 0.24, 0.03]	0	0.13	− 1.49	0.276	− 26.21	12.76	Stable
Quadriceps muscle strength on POD2	4	320	− 0.04 [− 0.18, 0.09]	0	0.52	0.5	0.669	− 20.91	26.36	Stable
Postoperative quadriceps muscle strength (overall)	4	640	− 0.07 [− 0.17, 0.02]	0	0.13					
Hospital stays (hour)	6	444	− 0.47 [− 0.78, − 0.16]	62	0.003	− 2.57	0.062	− 25.21	0.97	Stable
The incidence of PONV	6	440	0.62 [0.35, 1.09]	0	0.1	− 6.9	0.002	− 6.31	− 2.69	Stable

### *ACB* + *iPACK versus ACB: VAS scores*

**Fig. 3:**
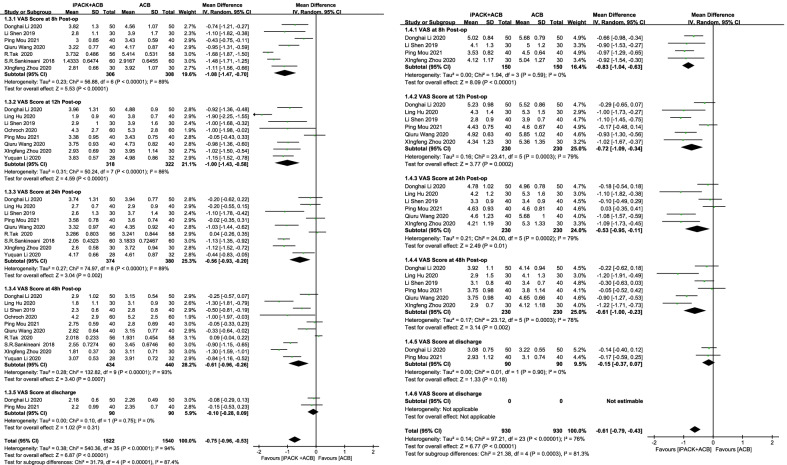
Forest plot of the postoperative VAS score at rest and with activity (CI: confidence interval; ACB: ultrasound-guided adductor canal blocks; and iPACK: ultrasound-guided local anesthetic infiltration of the interspace between popliteal artery and the capsule of posterior knee)

Figure 3 shows the VAS scores at different postoperative phases (8 h, 12 h, 24 h, 48 h, and at discharge) in the two groups. There are significant differences between the two groups in VAS scores at rest and with activity. VAS scores at rest: SMD = − 0.75, 95%CI [− 0.96, − 0.53], *I*^2^: 94%, *P* < 0.00001. VAS scores with activity: SMD = − 0.61, 95%CI [− 0.79, − 0.43], *I*^2^: 76%, *P* < 0.00001. Both findings are obviously in favor of the group of ACB + iPACK. When we divided them into subgroups according to different phrases, there was an apparent difference between the two groups at 8 h, 12 h, 24 h, and 48 h after the surgery. However, there was no significant difference between the two groups in comparing VAS at discharge (Fig. 3). Table 2 shows some extent of publication bias on the results of VAS score at rest at 12 h and 48 h and VAS score with activity at 48 h. Sensitivity analysis was also conducted by trim-and-fill analysis, and all results are unchangeable. This analysis confirmed the stability of the results (Table 2).

**Fig. 4:**
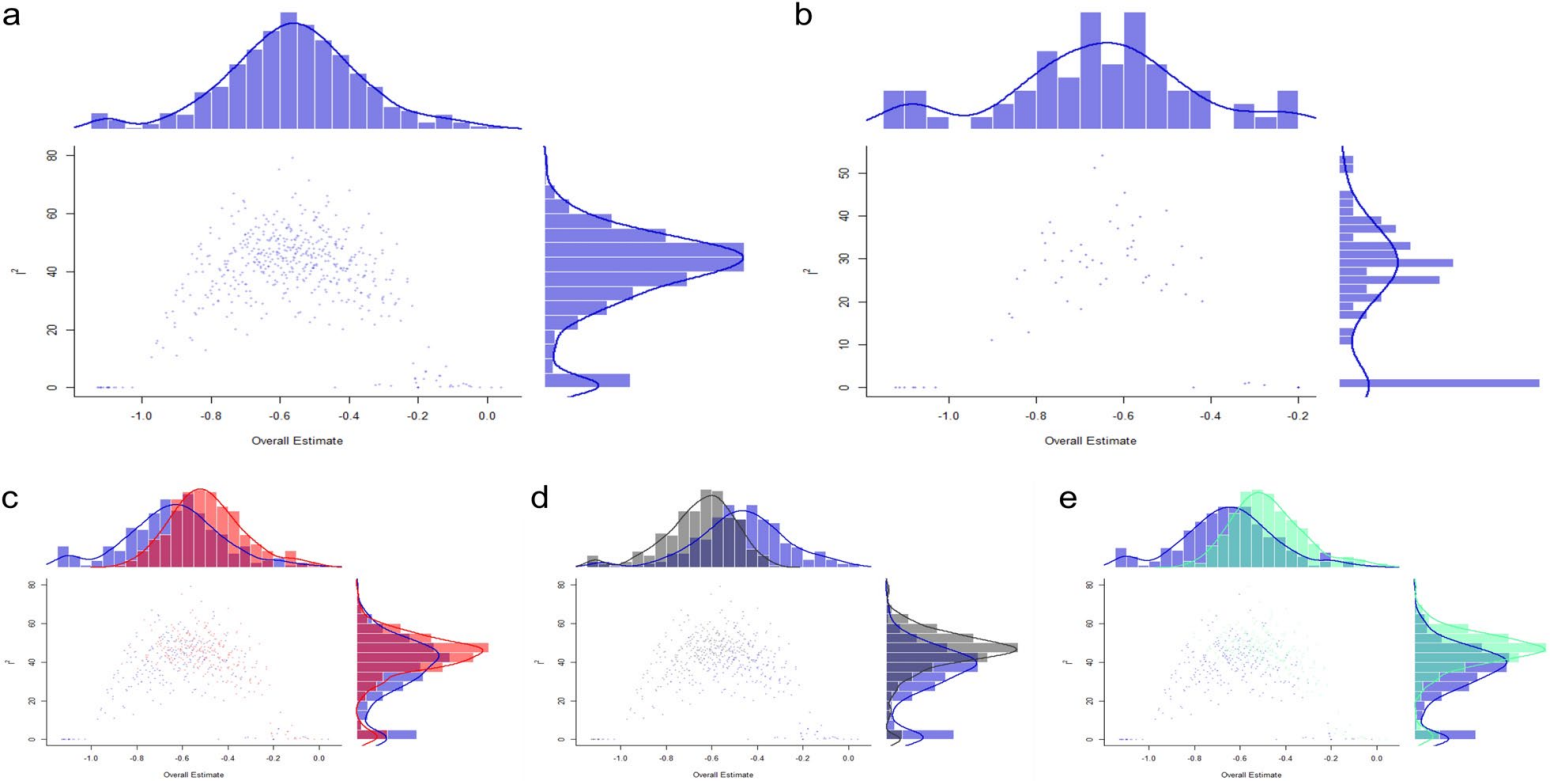
GOSH analysis based on the 24-h VAS scores at rest (**a**: the heterogeneity and overall effect before exclusion; **b**: the heterogeneity and overall effect after excluding the study of Ping Mou [26], Sankineani [21], and Tak [19]; **c**: the heterogeneity and overall effect after excluding the study of Ping Mou [26]; **d**: the heterogeneity and overall effect after excluding the study of Sankineani [21]; and **e**: the heterogeneity and overall effect after excluding the study of Tak [19].)

Due to the significant heterogeneity of the results, we performed subgroup analysis, meta-regression, and GOSH analysis based on the 24-h VAS score at rest. The GOSH analysis showed that no matter which literature was excluded, the heterogeneity did not change significantly, and the overall effect did not change significantly before and after exclusion (Fig. 4). It shows that although the heterogeneity is significant, the results are stable.

**Fig. 5:**
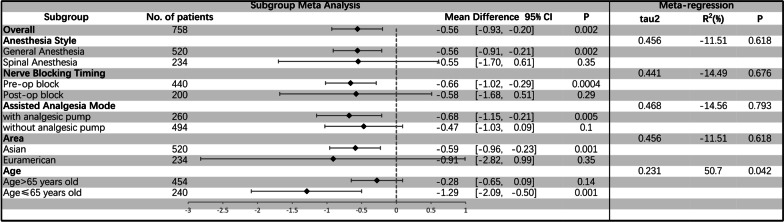
Subgroup meta-analysis and meta-regression analysis based on the 24-h VAS scores at rest (CI: confidence interval; Pre-op: preoperative; and Post-op: postoperative)

At the same time, we divided the subgroups according to different aspects of the study design (Anesthesia Style/Nerve blocking Timing/Assisted Analgesia Mode/Area/Patients’ Age). Figure 5 shows the results of subgroup meta-analysis and meta-regression. When we did a subgroup meta-analysis, we found that these factors affect the analysis results to a certain extent. There are more obvious differences between the two groups in those studies on general anesthesia, nerve block before the operation, postoperative analgesia pump, patients older than 65 years old, and studies in Asia (Fig. 5). However, the meta-regression results show that the patients’ age is the primary source of significant heterogeneity (*P* = 0.042).

### *ACB* + *iPACK versus ACB: postoperative cumulative morphine consumption*

**Fig. 6:**
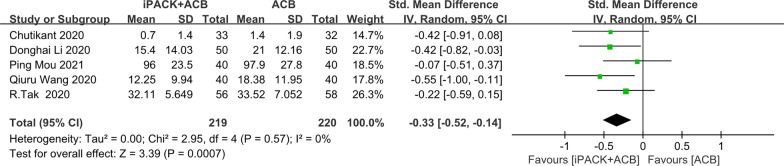
Forest plot of postoperative cumulative morphine consumption (CI: confidence interval; ACB: ultrasound-guided adductor canal blocks; and iPACK: ultrasound-guided local anesthetic infiltration of the interspace between popliteal artery and the capsule of posterior knee)

Among 14 included studies, 5 studies reported on cumulative morphine consumption (Fig. 6). These studies evaluated the mean difference in postoperative cumulative morphine consumption in 219 patients under the treatment of ACB + iPACK versus 220 patients under the treatment of ACB. A differential was discovered to support ACB + iPACK Group (SMD: − 0.33, 95%CI: [− 0.52, − 0.14], *P*: 0.0007, *I*^2^: 0%,).

### *ACB* + *iPACK versus ACB: postoperative range of knee movement (ROM)*

**Fig. 7:**
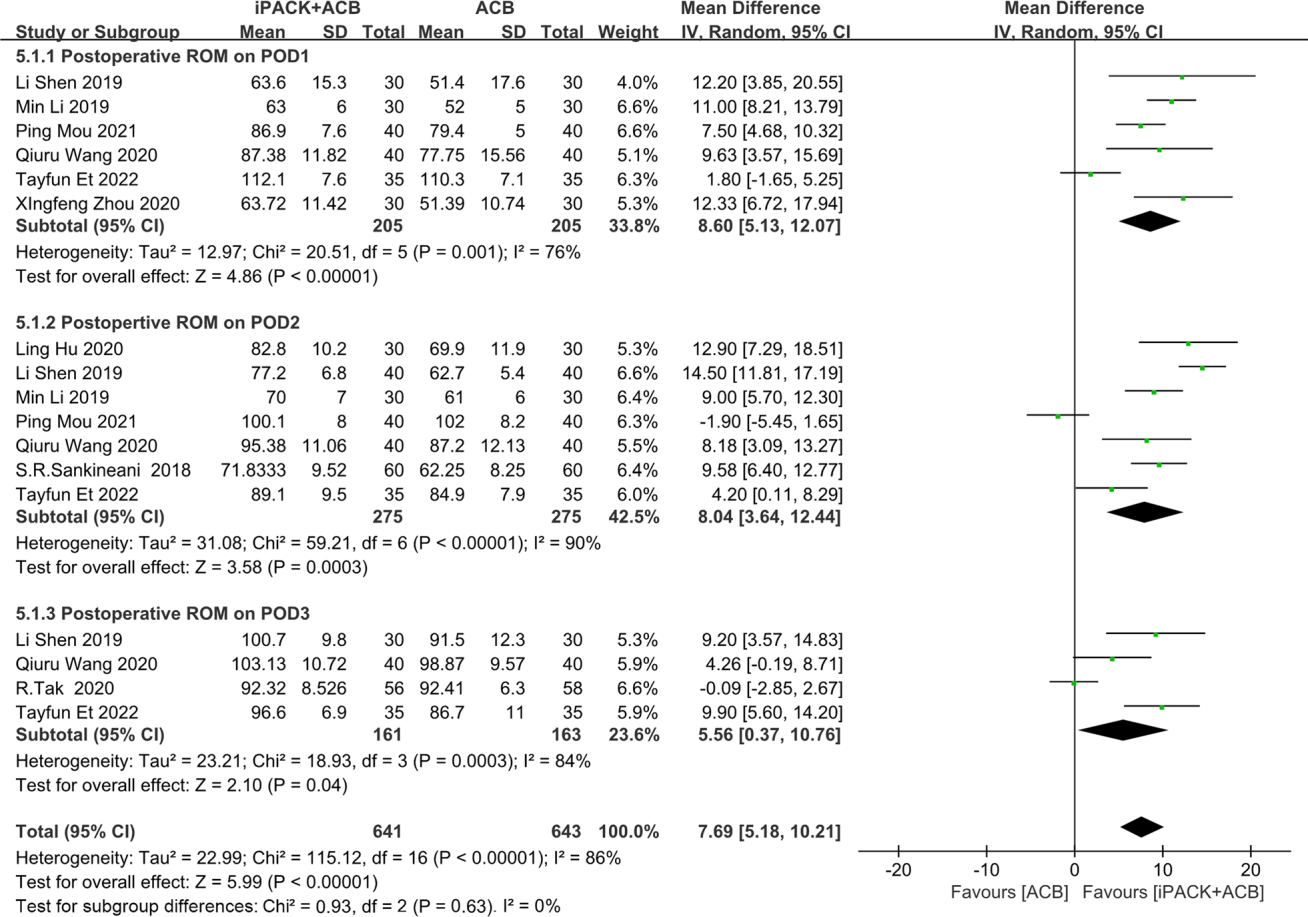
Forest plot of postoperative range of knee movement (CI: confidence interval; ACB: ultrasound-guided adductor canal blocks; and iPACK: ultrasound-guided local anesthetic infiltration of the interspace between popliteal artery and the capsule of posterior knee)

We included seven studies in the meta-analysis on the postoperative range of knee movement. Figure 7 shows an obvious difference between the two groups based on 1284 patients, which was obviously in favor of the group of ACB + iPACK (SMD: 7.69, 95%CI: [5.18, 10.21], *P* < 0.00001, *I*^2^:86%). At the same time, these studies are dissected into three subgroups by the differential time points after the surgery. There were statistically significant differences between the patients in the ACB + iPACK Group and ACB Group on ROM of POD1, POD2, and POD3 (Fig. 7).

### *ACB* + *iPACK versus ACB: TUG test*

**Fig. 8:**
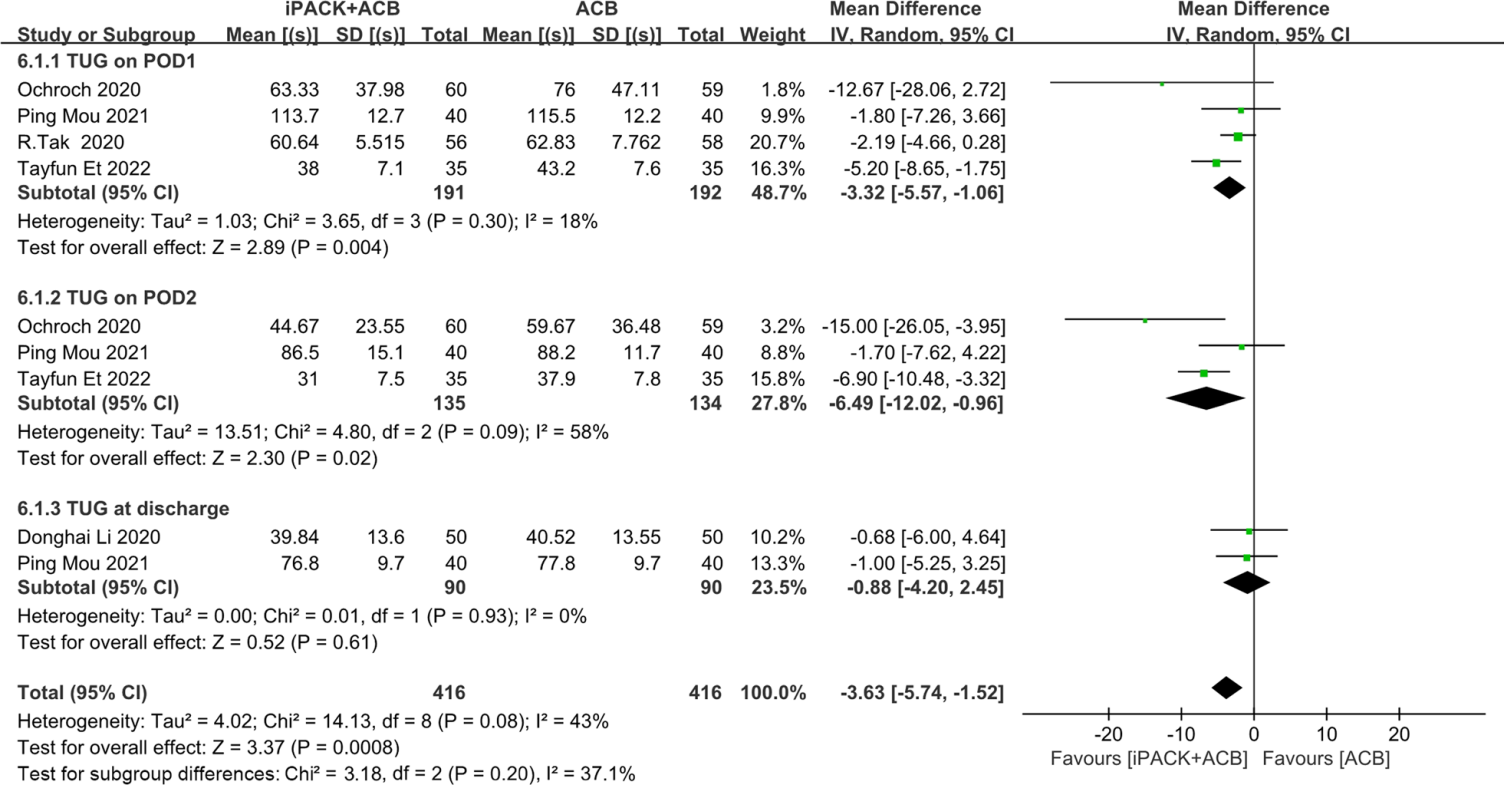
Forest plot of TUG test (CI: confidence interval; ACB: ultrasound-guided adductor canal blocks; and iPACK: ultrasound-guided local anesthetic infiltration of the interspace between popliteal artery and the capsule of posterior knee)

There is an obvious difference between the two groups based on 832 patients (Fig. 8). The finding was obviously in favor of the group of ACB + iPACK (SMD: − 3.63, 95%CI: [− 5.74, − 1.52], *P*: 0.0008, *I*^2^ = 43%). At the same time, these studies are dissected into three subgroups under the differential time points after the surgery. Four studies evaluated TUG one day after the surgery (Fig. 8). A differential was discovered to support ACB + iPACK Group (SMD: − 3.32, 95%CI: [− 5.57, − 1.06], *P*: 0.004, *I*^2^: 18%). Three explorations evaluated TUG on two days after the surgery (Fig. 8). There was a significant difference favoring the group of ACB + iPACK (SMD: − 6.47, 95% CI: [− 12.02, − 0.96], *P*: 0.02, *I*^2^: 58%,). The two groups presented no difference at discharge, according to two studies. (SMD: − 0.88, 95%CI: [− 4.20, 2.45], *P*: 0.61, *I*^2^: 0%) (Fig. 8).

### *ACB* + *iPACK versus ACB: postoperative walk distance*

**Fig. 9:**
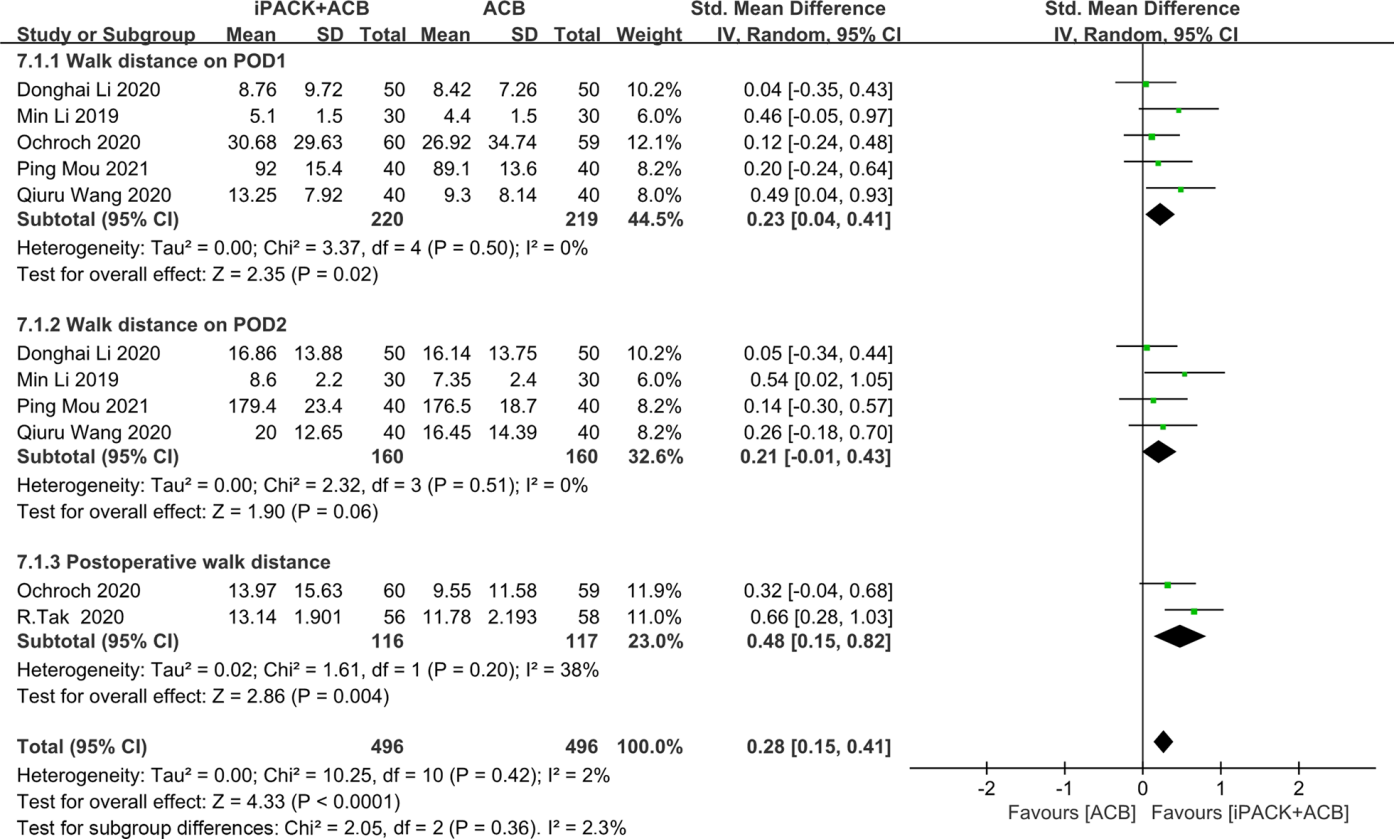
Forest plot of postoperative walk distance (CI: confidence interval; ACB: ultrasound-guided adductor canal blocks; and iPACK: ultrasound-guided local anesthetic infiltration of the interspace between popliteal artery and the capsule of posterior knee)

We included six studies in the meta-analysis on postoperative walk distance (Fig. 9). There is an obvious difference between the two groups based on 992 patients. The finding favors the group of ACB + iPACK (SMD: 0.28, 95%CI: [0.15, 0.41], *P* < 0.00001, *I*^2^: 2%) (Fig. 9). These studies are divided into three subgroups (one day/two days/cumulative walk distance after the surgery). Five studies evaluated the mean difference one day after surgery (Fig. 9). An obvious differential was found in favor of ACB + iPACK Group (SMD: 0.23 95%CI: [0.04, 0.41], *P*: 0.02, *I*^2^: 0%). Four studies assessed the mean difference on POD2, and no difference was found (SMD: 0.21 95%CI: [− 0.01, 0.43], *P*: 0.06, *I*^2^: 0%) (Fig. 9). Two studies assessed the mean difference in cumulative walk distance, and an apparent differential was found favoring the ACB + iPACK Group (SMD: 0.48, 95% CI: [0.15, 0.82], *P*: 0.004, *I*^2^ = 38%) (Fig. 9).

### *ACB* + *iPACK versus ACB: postoperative quadriceps muscle strength*

**Fig. 10:**
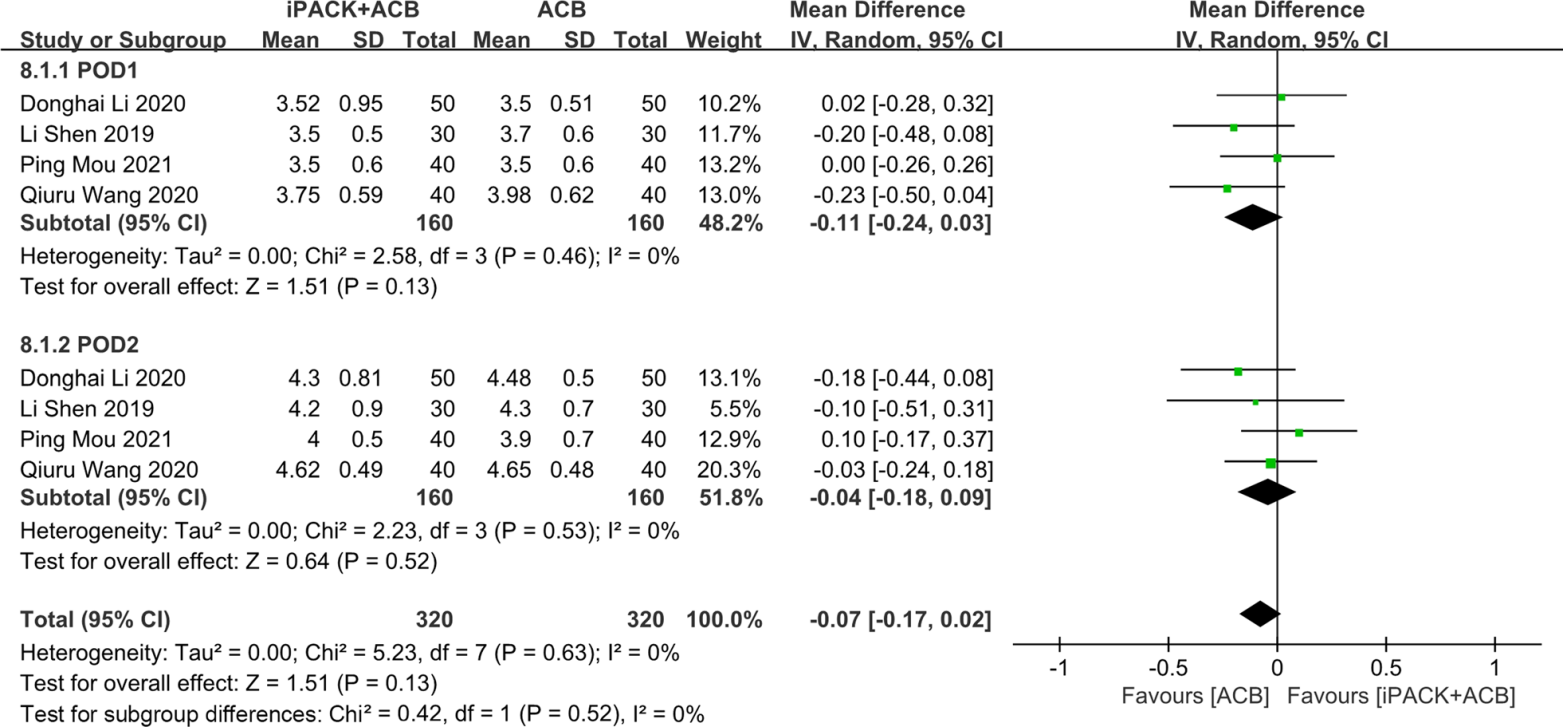
Forest plot of postoperative quadriceps muscle strength (CI: confidence interval; ACB: ultrasound-guided adductor canal blocks; and iPACK: ultrasound-guided local anesthetic infiltration of the interspace between popliteal artery and the capsule of posterior knee)

We included four studies in the meta-analysis on postoperative quadriceps muscle strength (Fig. 10). No difference was found between the patients in ACB + iPACK Group and ACB Group, based on 640 patients (SMD: − 0.07, 95%CI: [− 0.17, 0.02], *P*: 0.13, *I*^2^: 0%). These researches are divided into two subgroups, and no obvious difference could be seen between the two groups in the subgroup meta-analysis.

### *ACB* + *iPACK versus ACB: hospital stays and the incidence of PONV*

**Fig. 11:**
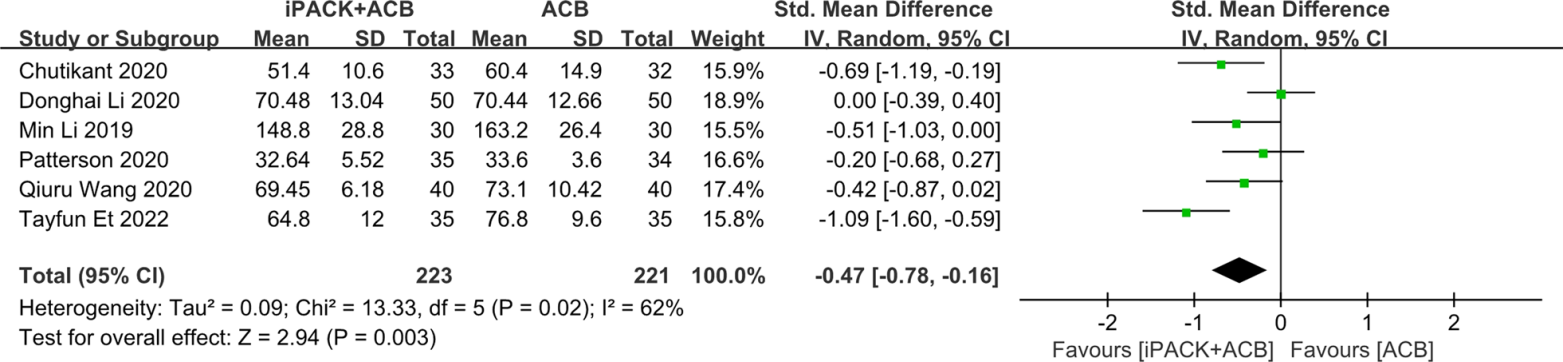
Forest plot of hospital stays (CI: confidence interval; ACB: ultrasound-guided adductor canal blocks; and iPACK: ultrasound-guided local anesthetic infiltration of the interspace between popliteal artery and the capsule of posterior knee)

**Fig. 12:**
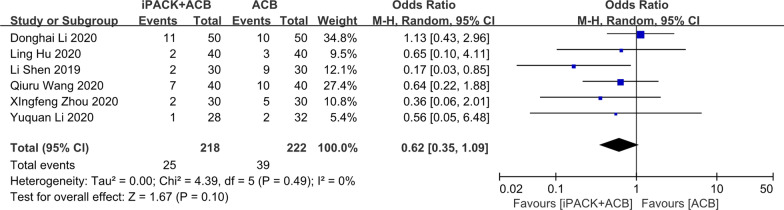
Forest plot of the incidence of PONV (CI: confidence interval; ACB: ultrasound-guided adductor canal blocks; and iPACK: ultrasound-guided local anesthetic infiltration of the interspace between popliteal artery and the capsule of posterior knee)

Among these 14 studies, six studies research patients' hospital stays (Fig. 11). There is an apparent difference between the two groups based on 444 patients supporting the ACB + iPACK Group (SMD: − 0.47, 95%CI: [− 0.78, − 0.16], *P*: 0.003, *I*^2^: 62%). There is no difference between the patients in ACB + iPACK Group and ACB Group on the incidence of PONV (OR: 0.62, 95%CI: [0.35, 1.09], *P*: 0.1, *I*^2^: 0%) (Fig. 12).

## Discussion

The research expounds on the all-sides meta-analysis involving all clinical trials to investigate whether iPACK block added to ACB could improve analgesia outcomes after TKA. Despite that, a meta-analysis investigated the same question before [27]. However, it did not cover all clinical trials, and there are many other aspects this literature did not involve, which has left some questions open on these aspects. Furthermore, several different findings were found in our study. In our meta-analysis, the critical finding is that the addition of iPACK did reduce postoperative VAS scores no matter whether the patients were at rest or with activity. Furthermore, the supplement of iPACK could reduce postoperative cumulative morphine consumption. In all, we consider that the addition of iPACK can effectively reduce patients’ postoperative pain and reduce the use of postoperative morphine consumption.

ACB, which offers analgesia to the peripatellar and intra-articular aspects of the knee joint, cannot reduce posterior knee pains. As a novel technique, iPACK block mainly targets terminal branches of the sciatic nerve in the knee joint’s posterior capsule [28]. The point of injection is located in the position of the distal popliteal fossa at the level of the femoral condyle, in which the popliteal plexus is formed previous to the entry to the knee joint’s back [29, 30]. On the plane, the common peroneal nerve, which extends outward from the surface of the posterior capsule, makes an entire separation out of the tibial nerve. In addition, the functions made by the tibial nerve motor have already been primarily preserved as well [31, 32]. These studies are consistent with our results.

According to our results, the addition of iPACK did improve the activity performance in some aspects. The addition of iPACK increases the patients’ cumulative walk distance after the surgery and shorter the hospital stays of patients. Besides, the patients in the group of iPACK + ACB performed better in the TUG test and postoperative ROM. However, the two groups took on no difference in postoperative quadriceps muscle strength.

Postoperative ROM is a vital outcome evaluation index after TKA and reflects the related muscle strength of the knee [33]. TUG test and the postoperative walking distance directly reflect the mobility of lower limbs [34]. The patients in the group of iPACK + ACB performed better in these three aspects, indicating that the addition of iPACK can improve the activity performance of patients. The motor nerve of the quadriceps muscles is mainly the femoral nerve, and the iPACK block is mainly aimed at the terminal branch of the sciatic nerve in the posterior capsule of the knee joint. Thus, the addition of iPACK block does not affect the movement of the femoral nerve. Edmund Chan did a narrative review through 35 articles and mentioned that ACB and iPACK block would not increase the nerve block of quadriceps muscles, which is also consistent with our results [35].

Among our results, the heterogeneity of VAS score meta-analysis is considered significant, and we found this apparent heterogeneity does not originate from individual studies through performing GOSH analysis. At the same time, the GOSH analysis and sensitivity analysis manifest that although the heterogeneity is significant, the results are stable. That is, the addition of iPACK did reduce postoperative VAS scores no matter whether the patients were at rest or with activity. Meanwhile, the subgroup analysis shows that anesthesia style, nerve block timing, research area, patients’ age, and perioperative analgesia strategy affect the heterogeneity of the results to a certain extent. And meta-regression analysis shows that the patients’ age is the main origin of the significant heterogeneity. Besides, most results are stable and believable through performing sensitivity analysis. So iPACK block has a remarkable effect in relieving posterior knee pain with neither postoperative functional recovery nor adverse complications.

There are some limits in our study. In spite of several publications, exploring protocols and outcomes are featured by discrepancies (e.g., perioperative analgesia strategy), impeding statistical analysis and leading to considerable heterogeneity.

## Conclusion

In conclusion, the study shows that iPACK integrated with ACB proves a hopefully bright technique that can upgrade pain management during the immediate postoperative period with no influence on motor activity. The addition of iPACK lowers postoperative VAS scores, cumulative morphine consumption, and hospital stays. Meanwhile, the addition of iPACK improves postoperative patients’ activity performance without extra side effects.

## Data Availability

All data included in this study are available upon request by contact with the corresponding author.

## Abbreviations

iPACK: The popliteal artery and the posterior knee capsule have been given interspace local anesthetic infiltration
ACB: Adductor canal block
TKA: Total knee arthroplasty
VAS: Visual analog scale
GOSH: Graphical display of study heterogeneity
POD1: The first day after the surgery
POD2: The second day after the surgery
POD3: The third day after the surgery
RCTs: Randomized controlled trials
TUG: Timed Up and Go
PONV: Postoperative nausea and vomiting
GRADE: Grading of recommendations, assessment, development and evaluation
RR: Relative risk
OR: Odds ratio
CI: Confidence intervals
RoB: Risk of bias
SMD: Standard mean difference
ROM: Postoperative range of knee movement
